# Comment on Al Barri et al. Evaluation of Refractive Predictive Accuracy in Intraocular Lens Power Calculations: A Comparative Study of Swept-Source Optical Coherence Tomography and Optical Low-Coherence Interferometry. *J. Clin. Med.* 2025, *14*, 1201

**DOI:** 10.3390/jcm14228011

**Published:** 2025-11-12

**Authors:** Domenico Mucci, Benedetta Cioffi

**Affiliations:** Ophthalmological Unit, Department of Medicine, Surgery and Dentistry, Scuola Medica Salernitana, University of Salerno, Via S. Allende, Baronissi, 84081 Salerno, Italy; benedettacioffi00@gmail.com

We read with great interest the article titled “Evaluation of Refractive Predictive Accuracy in Intraocular Lens Power Calculations: A Comparative Study of Swept-Source Optical Coherence Tomography and Optical Low-Coherence Interferometry” by Al Barri et al. [1]. We commend the authors for addressing such a relevant and clinically significant topic: the comparative performance of biometry devices in IOL power calculation.

While we truly appreciate the effort and the insights offered, we would like to respectfully share a few observations that may affect the interpretation and generalizability of their findings.

First, we noted that their study included 170 eyes from 102 patients, without reporting any correction for inter-eye correlation. Considering both eyes from the same patient as independent observations can increase sample size and introduce bias in statistical significance testing, particularly when evaluating subtle differences in refractive outcomes. Statistical approaches such as generalized estimating equations (GEE) or mixed-effects modeling would be more appropriate to account for this choice [2]; without it, the risk of Type II error is high.

Additionally, the unequal sample sizes between the swept-source Optical Coherence Tomography (OCT) group (*n* = 133 eyes) and the Optical Low-Coherence Interferometry (OLCI) group (*n* = 37 eyes) weaken the statistical power for between-group comparisons. Although bootstrapping was applied, the markedly smaller cohort size in the OLCI arm limits the robustness of between-group comparisons, particularly in the toric IOL subgroup, which comprised only 10 cases in the OLCI group. This limited sample size weakens the reliability of conclusions regarding astigmatism correction and refractive accuracy.

We also observed that the retrospective nature of this study and the non-randomized assignment of biometry device—based on clinical workflow and device availability—introduce potential selection bias. Recent studies on IOL power prediction have emphasized the importance of stratified group design, axial length (AL)-based subgrouping, and formula-device pairing to enhance the validity of outcome comparisons. For instance, the studies by Cione et al. employed larger and more homogeneous populations across well-defined biometric categories, while also incorporating both historical and contemporary datasets to ensure reproducibility and generalizability [3,4].

Although both groups used the Barrett Universal II formula, the authors do not report whether lens constants were individually optimized for each device. As you are aware, device-specific constant optimization is crucial for achieving accurate refractive outcomes. It was demonstrated that the concept of adjusting ULIB-optimized constants via subtraction can reduce systematic prediction error in post-refractive cases [3]. This constant modulation methodology significantly improved both median absolute error and the percentage of eyes within ±0.50 D of prediction error—metrics that were not fully explored in this analysis.

The relatively small number of eyes in the OLCI group, especially when analyzing subgroups, limits the robustness of conclusions. For instance, only 10 toric IOLs were implanted in the OLCI group compared to 104 in the SS-OCT group.

Differences in axis alignment or astigmatism correction efficacy should be interpreted with caution due to this imbalance.

Additionally, this study does not provide stratification or adjustment based on AL, despite well-established evidence that prediction errors vary significantly with AL, particularly in long eyes [4]. This approach is increasingly being recognized as a standard for biometry validation studies.

We also noted the absence of a priori sample size calculation or detailed assessment of the error distribution. Both aspects are now routinely included in IOL power prediction studies to enhance transparency and reproducibility [3,4,5].

In conclusion, while the study contributes valuable data to the evolving landscape of optical biometry, considering the above methodological aspects could further enrich the interpretation of the findings. Future investigations would benefit from prospective designs, randomization, lens constant harmonization, and subgroup analysis by AL range to enable a more precise assessment of device-specific performance.
